# Slowly Progressive and Painless Thoracic Aortic Dissection Presenting with a Persistent Fever in an Elderly Patient: The Usefulness of Combined Measurement of Biochemical Parameters

**DOI:** 10.1155/2013/498129

**Published:** 2013-06-17

**Authors:** Shunsuke Yamada, Masanori Tokumoto, Toshiaki Ohkuma, Yasuo Kansui, Yoshinobu Wakisaka, Yuji Uchizono, Kazuhiko Tsuruya, Takanari Kitazono, Hiroaki Ooboshi

**Affiliations:** ^1^Division of Internal Medicine, Fukuoka Dental College, 2-15-1 Tamura, Sawara-Ku, Fukuoka 814-0193, Japan; ^2^Department of Medicine and Clinical Science, Graduate School of Medical Sciences, Kyushu University, Fukuoka 812-8582, Japan; ^3^Department of Integrated Therapy for Chronic Kidney Disease, Graduate School of Medical Sciences, Kyushu University, Fukuoka 812-8582, Japan

## Abstract

Aortic dissection is a fatal medical condition that requires urgent diagnosis and appropriate intervention. Because acute aortic dissection often manifests as sudden onset excruciating chest pain, physicians can easily reach a proper diagnosis. However, some patients with aortic dissection present with varied clinical manifestations without exhibiting typical chest pain, leading to a delayed diagnosis and possible fatality. We herein present the case of an elderly subject with a fever of unknown origin who was ultimately diagnosed with aortic dissection. In the present case, a negative procalcitonin test, increased D-dimer and serum creatinine phosphokinase-BB levels, and reelevation of the CPR level led us to the correct diagnosis.

## 1. Introduction

Aortic dissection typically presents with excruciating chest pain, and the correct diagnosis can often be obtained using imaging techniques, including contrast-enhanced computed tomography [1]. However, some patients with painless aortic dissection present with a variety of symptoms and manifestations such as myocardial infarction, aortic regurgitation, intrathoracic hemorrhage, and fever of unknown origin (FUO), resulting in a delayed diagnosis and the possibility of fatality [2–4].

We herein describe the case of an elderly patient presenting with a persistent fever who was ultimately diagnosed with a painless thoracoabdominal aortic dissection. In the present case, a high serum C-reactive protein (CRP) level with a negative procalcitonin test and increased serum creatinine kinase BB isozyme (CK-BB) and blood D-dimer levels were helpful in diagnosing slowly progressive and painless aortic dissection. 

## 2. Case Report

An 86-year-old female was hospitalized due to anorexia and a persistent fever lasting for the previous three weeks. She had been receiving regular follow-up care for hypertension and an old brain infarction at our hospital. She had undergone endovascular stent grafting for a thoracic and abdominal aortic aneurysm six months prior to admission. At the last visit three weeks prior to admission, she underwent regular computed tomography, which revealed an aortic aneurysm with stent grafting measuring 55 mm in diameter. This was almost identical to the diameter observed just after stent insertion. At that point, she had no complaints, and no inflammatory responses were observed on a biochemistry analysis. 

**Figure 1:**
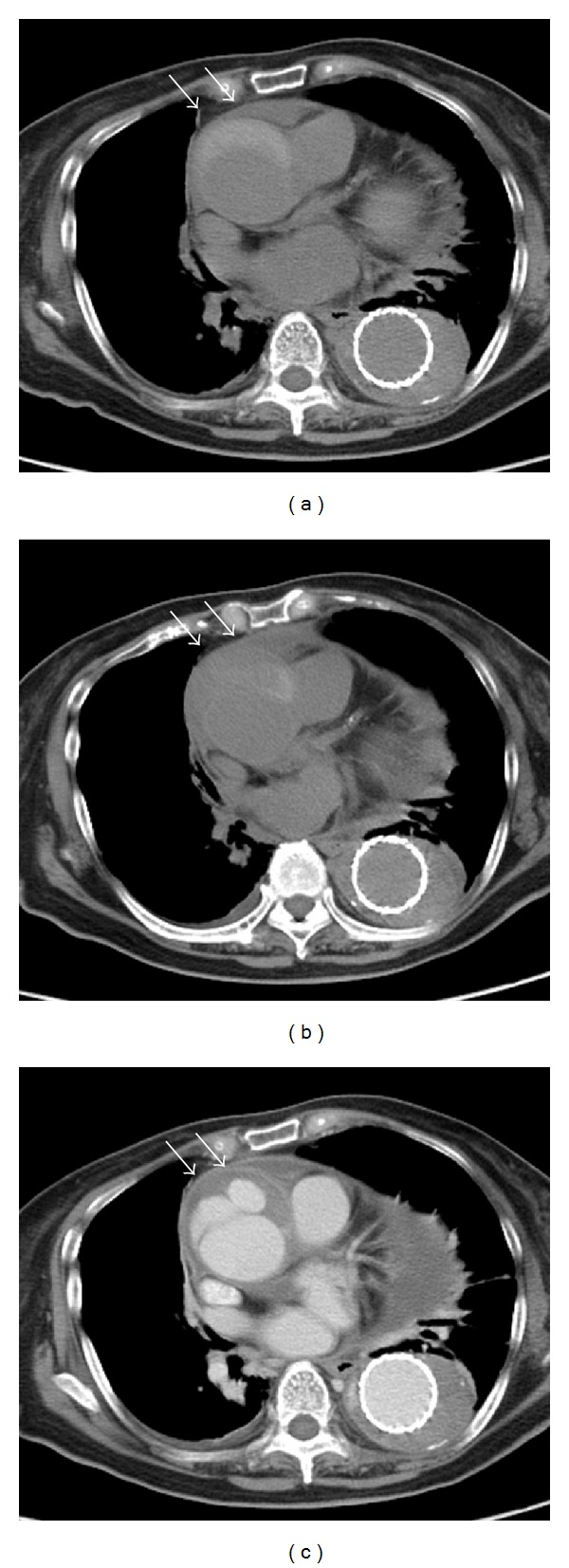
Computed tomography of the chest. (a) Plain computed tomography (CT) performed on the first day. The density of the false lumen (white arrows) of the ascending aorta was homogenous and higher than that of the true lumen and that of the false lumen of the descending aorta, indicating relatively newly developed thrombosis. The maximum diameter of the ascending aorta was 55 mm. (b) Plain CT performed on the 27th day. The density of the false lumen of the ascending aorta was partially heterogeneous compared to that observed on the first day. (c) Contrast-enhanced CT performed on the 29th day. The false lumen of the ascending aorta was also enhanced by contrast medium, directly indicating ongoing aortic dissection. The amount of pericardial effusion was also increased. The maximum diameter of the ascending aorta was enlarged (60 mm), indicating an increased risk for rupture of the ascending aorta.

On hospitalization, the patient was alert. Her blood pressure was 148/90 mmHg, her heart rate was 90 beats/min, her body temperature was 37.8°C, and her respiratory rate was 12 breaths/min. A physical examination revealed weak inspiratory coarse crackles in the left lower lobe. The patient's biochemical parameters and urinary findings are summarized in Table 1. The white blood cell count was 7600/*μ*L with 80.8% neutrophils. The CRP level was 197 nmol/L. The levels of fibrin degradation product (FDP) and D-dimer were not measured at that time. Laboratory tests related to autoimmune diseases were all negative. Blood cultures for bacteria, the levels of endotoxin and serum **β**-D-glucan, and an interferon-gamma release assay for tuberculosis were all negative. Electrocardiogram was normal. Echocardiography revealed a moderate level of pericardial effusion with a preserved left ventricular ejection fraction and mild tricuspid regurgitation. Plain computed tomography disclosed a moderate level of pericardial effusion, infiltration in the left lower lobe, and a small amount of bilateral hydrothorax and the endovascular stent graft from the middle part of the aortic arch to the upper part of the descending abdominal aorta. The false lumen of the ascending aorta exhibited a relatively high density compared to the abdominal aorta (Figure 1(a)). However, the diameter of the ascending aorta was 55 mm, identical to that observed three weeks prior to admission. Although we also considered the possible involvement of a stent infection, we were unable to confirm this diagnosis using positron emission tomography or 67Ga scintigraphy; these modalities were not available at our hospital.

At that point, we were unable to determine the cause of the high serum CRP level, and we administered 2 g/day of sulbactam cefoperazone under a tentative diagnosis of pneumonia in the left lower lung. Because the result of the serum procalcitonin test performed on admission was negative, the antibiotic therapy was discontinued. Although antibiotic treatment was stopped, the serum CRP level continued to decrease, and the patient was discharged on the 11th day. On day 19, the serum CRP level was 32.4 nmol/L at the outpatient clinic. However, 15 days after discharge (the 27th day), the patient again developed an elevated serum CRP level (161 nmol/L) and high fever.

**Figure 2:**
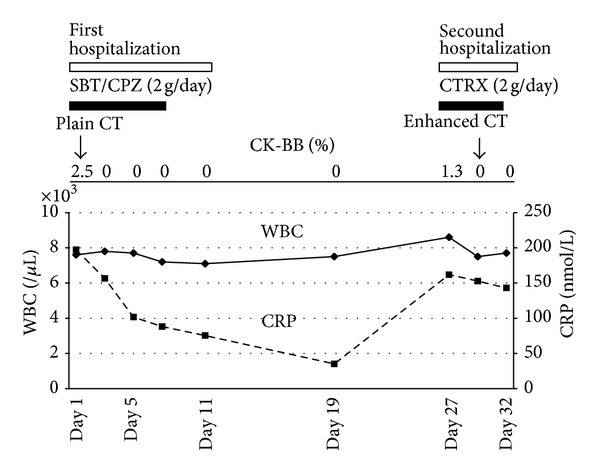
Clinical course during the first and second hospitalizations.  The serum level of CRP decreased after the first hospitalization. However, it increased again at the time of the second hospitalization and remained elevated in spite of the administration of antibiotics. The percentage of the serum CK-BB isozyme level exhibited an abrupt increase twice. CK-BB creatinine kinase-BB, CT computed tomography, CTRX ceftriaxone, CRP C-reactive protein, SBT/CPZ sulbactam cefoperazone, and WBC white blood cell count.

On the second hospitalization, the serum procalcitonin test was again negative. The FDP level was 37 mg/L (*N*; <10), the D-dimer level was 96.0 nmol/L (*N*; <3.0), and the fibrinogen level was 19.1 *μ*mol/L (*N*; 5.8–11.8). CK-BB isozyme was also increased (1.3%). Contrast-enhanced computed tomography was performed on the 27th day, which revealed enlargement of the diameter of the aorta (from 55 mm to 60 mm) and partial enhancement of the false lumen of the ascending aorta (Figures 1(b) and 1(c)). Further, the pericardial effusion had further increased compared to that observed on the first day. The patient was finally diagnosed as having dissection of the ascending aorta, in particular, Stanford type A aortic dissection with thrombosis of the false lumen of the ascending aorta (Figures 1(b) and 1(c)). She was immediately transferred to the university hospital and underwent total aortic arch replacement with an artificial graft. After the operation, the patient's serum CRP level became normal, indicating that the inflammation of the aortic wall was the cause of the high serum CRP level with the negative procalcitonin test (Figure 2).

## 3. Discussion

There are several points to be emphasized in the present study. First, slowly progressive aortic dissection, particularly painless aortic dissection, is easily underestimated, resulting in a delayed diagnosis. Second, aortic dissection is involved in the development of nonbacterial arterial inflammation, which is determined according to a high serum CRP level with a negative procalcitonin test. Third, the CK-BB level can be used as a supportive marker to detect aortic dissection. Fourth, increased blood D-dimer and FDP levels are helpful when CT does not clearly show the typical findings of aortic dissection. Fifth, the biphasic elevation of inflammatory markers indicates redissection of the aorta. 

As evidenced by the present case, some patients with aortic dissection present with a persistent fever and an elevated inflammatory response that are considered to be FUO [2–4]. Recent clinical and experimental studies have revealed that aortic dissection is mediated by inflammation in the adventitia and media of the aorta, leading to degradation of the extracellular matrix, including elastin, and tearing in the medial layer [5–7]. According to histological analyses, inflammatory cells such as macrophages and lymphocytes are recruited to the aortic media, and matrix metalloproteinase 9 is involved in the degradation of elastin and other components of the extracellular matrix [8]. Collectively, serum inflammatory markers, including CRP, are often elevated in these patients. Interestingly, one observational study showed that reelevation of the serum CRP level is a useful marker of redissection of the aorta [9]. The authors speculated that local thrombogenesis triggers inflammatory cytokine release, leading to an increased production of CRP [9]. In the present case, the high CRP level observed on the first and second admissions could be attributed to ongoing dissection of the ascending aorta. Furthermore, recent clinical studies have shown that the level of CK-BB isoenzyme is elevated in the acute phase of aortic dissection [10]. This result is consistent with the present case in that the CK-BB level was abruptly increased on the second day of the first admission and on the first day of the second admission, indicating a flare up in the activity of the ascending aortic dissection around these time points [10]. These results indicate that the patient could have developed insidious aortic dissection during the first hospitalization, although we did not determine the definitive findings using contrast-enhanced computed tomography.

Procalcitonin is produced in all cells in response to interleukin-1, interleukin-6, and tumor necrosis factor and is inhibited by interferon gamma [11]. Because the serum procalcitonin level is increased exclusively under bacterial infections and some other limited medical conditions (fungal infections and other types of inflammation), it can be used to make the differential diagnosis between bacterial and viral infections. A meta-analysis showed that the sensitivity and specificity in patients with sepsis are 77% and 79%, respectively, although these values depend on the examined cohorts [12]. In addition, viral infections are often accompanied by high serum interferon gamma levels, which inhibit the production of serum procalcitonin [13]. In contrast, the serum CRP level cannot be used to differentiate bacterial infections from other causes and is therefore less useful for diagnosing systemic inflammation [14]. In the present case, the discrepancy between the high serum CRP level and the negative test result for procalcitonin indicated the presence of nonbacterial inflammation, which led to the search for other forms of inflammation. In this regard, when patients suffer from inflammation, as evidenced by a high CRP level, the procalcitonin level can be used to distinguish bacterial infections from other pathogeneses with relatively high specificity, enabling prompt and correct diagnosis of patients. In this regard, a negative procalcitonin test with a high C-reactive protein level is a new and good diagnostic marker of aortic dissection, especially the painless type. 

Recent clinical studies have revealed that aortic dissection is often accompanied by high levels of blood FDP and D-dimer, indicating the presence of local thrombosis and thrombolysis and aiding in reaching the correct diagnosis [15]. Clinical guidelines for acute aortic dissection state that a D-dimer level <500 ng/mL exhibits 99.5% negative predictive value for acute aortic dissection in patients with chest pain, while a D-dimer level >1,600 ng/mL exhibits 95% positive predictive value [16]. Indeed, in the present case, the D-dimer level was greater than 1,600 ng/mL (16,700 ng/mL), being compatible with the typical laboratory findings of acute aortic dissection. Although the usefulness of measuring the D-dimer level is confirmed only when aortic dissection occurs within 6 to 24 hours, it can be of great help in making the differential diagnosis of acute-onset chest pain.

We are aware that there are several putative limitations in the present study. First, we did not measure either FDP or D-dimer during the first hospitalization. If the aortic dissection was ongoing during the first hospitalization, repeated measurement of the blood FDP and D-dimer levels could have been helpful in determining the timing of the onset of aortic dissection. Second, only plain computed tomography was performed during the first hospitalization. If enhanced computed tomography had been performed, the aortic dissection could have been detected much earlier. Third, measuring the serum CK-BB level remains a preliminary laboratory test, and clinical application of this parameter should be used cautiously for the time being.

In summary, we herein presented the case of an elderly patient with a persistent fever, a high serum CRP level, and a negative procalcitonin test who was ultimately diagnosed with painless aortic dissection of the ascending aorta. Physicians should remember that chronic aortic dissection can manifest as a sustained fever and a high serum CRP level without typical chest pain. In addition, they should be aware of the usefulness of determining the serum procalcitonin, CK-BB, and D-dimer levels concomitantly when making the differential diagnosis of persistent high fever in patients with a history of prior stent insertion for aortic aneurysm.

## Figures and Tables

**Table 1:** Laboratory data obtained on the first admission.

Complete blood count	
White blood cell count, /*μ*L	7600
Hemoglobin, g/dL	10.8
Platelets, ×10^4^/*μ*L	22.1
Biochemistry	
Total protein, g/L	58
Albumin, g/L	34
Total bilirubin, *μ*mol/L	13.7
Aspartate aminotransferase, U/L	45
Alanine aminotransferase, U/L	51
Alkaline phosphatase, U/L	389
*γ*-GTP, U/L	46
Lactate dehydrogenase, U/L	183
Creatine phosphokinase, U/L	419
Blood urea nitrogen, mmol/L	10.0
Creatinine, *μ*mol/L	81.4
Uric acid, *μ*mol/L	375
Glucose, mmol/L	6.6
Hemoglobin A1c, %	5.6
Brain natriuretic peptide, ng/L	149
Ferritin, pmol/L	1742
Urinalysis and urinary sediment	
pH (dipstick)	6.5
Specific gravity (dipstick)	1.025
Protein (dipstick)	1+
Hematuria (dipstick)	1+
Bilirubin	Negative
Ketone	Negative
Immunology	
Antinuclear antibody	×40
AntiSmith antibody	Negative
Anti-double-stranded DNA antibody	Negative
Soluble interleukin-2 receptor, U/mL	580
C3, g/L	1.62
C4, g/L	0.46
IgG, g/L	10.2
IgA, g/L	4.2
IgM, g/L	0.8
Infection-related test	
*β*-D-Glucan, pg/mL (normal; <0.5)	<0.5
Endotoxin, pg/mL (normal; <11.0)	<11.0
Procalcitonin, ng/mL (normal; <0.5)	0.15

DNA: deoxyribonucleic acid, GTP: glutamyl transpeptidase, Ig: immunoglobulin, and PT/INR: prothrombin time/international normalized ratio.

## References

[1] Braverman AC (2011). Aortic dissection: prompt diagnosis and emergency treatment are critical. *Cleveland Clinic Journal of Medicine*.

[2] Cheng C-C, Lin C-Y, Han C-L (2007). Intramural haematoma of the aorta presenting as fever of unknown origin. *Acta Cardiologica*.

[3] Miyairi T, Inaba H, Matsumoto J, Tanaka K, Kanda J, Suzuki M (1998). Dissecting aortic aneurysm presenting as pyrexia of unknown origin: report of a case. *Surgery Today*.

[4] Mc Keown PP, Campbell NP (1989). Pyrexia of unknown origin and aortic dissection. *International Journal of Cardiology*.

[5] Murray HW, Mann JJ, Genecin A, McKusick VA (1976). Fever with dissecting aneurysm of the aorta. *American Journal of Medicine*.

[6] Luo F, Zhou X-L, Li J-J, Hui R-T (2009). Inflammatory response is associated with aortic dissection. *Ageing Research Reviews*.

[7] Okina N, Ohuchida M, Takeuchi T (2013). Utility of measuring C-reactive protein for prediction of in-hospital events in patients with acute aortic dissection. *Heart and Vessels*.

[8] Sakakura K, Kubo N, Ako J (2010). Peak C-reactive protein level predicts long-term outcomes in type B acute aortic dissection. *Hypertension*.

[9] Makita S, Ohira A, Tachieda R (2000). Behavior of C-reactive protein levels in medically treated aortic dissection and intramural hematoma. *American Journal of Cardiology*.

[10] Suzuki T, Katoh H, Kurabayashi M, Yazaki Y, Nagai R (1997). Biochemical diagnosis of aortic dissection by raised concentrations of creatine kinase BB-isozyme. *The Lancet*.

[11] Delèvaux I, André M, Colombier M (2003). Can procalcitonin measurement help in differentiating between bacterial infection and other kinds of inflammatory processes?. *Annals of the Rheumatic Diseases*.

[12] Wacker C, Prkno A, Brunkhorst FM, Schlattmann P (2013). Procalcitonin as a diagnostic marker for sepsis: a systemic review and meta-analysis. *The Lancet Infectious Diseases*.

[13] Schuetz P, Albrich W, Mueller B (2011). Procalcitonin for diagnosis of infection and guide to antibiotic decisions: past, present and future. *BMC Medicine*.

[14] Simon L, Gauvin F, Amre DK, Saint-Louis P, Lacroix J (2004). Serum procalcitonin and C-reactive protein levels as markers of bacterial infection: a systematic review and meta-analysis. *Clinical Infectious Diseases*.

[15] Sodeck G, Domanovits H, Schillinger M (2007). D-dimer in ruling out acute aortic dissection: a systematic review and prospective cohort study. *European Heart Journal*.

[16] Suzuki T, Distante A, Zizza A (2009). Diagnosis of acute aortic dissection by D-dimer: the international registry of acute aortic dissection substudy on biomarkers (IRAD-bio) experience. *Circulation*.

